# Correlation of Global MicroRNA Expression With Basal Cell Carcinoma Subtype

**DOI:** 10.1534/g3.111.001115

**Published:** 2012-02-01

**Authors:** Christopher Heffelfinger, Zhengqing Ouyang, Anna Engberg, David J. Leffell, Allison M. Hanlon, Patricia B. Gordon, Wei Zheng, Hongyu Zhao, Michael P. Snyder, Allen E. Bale

**Affiliations:** * Department of Molecular, Cellular and Developmental Biology, Yale University, New Haven, CT 06520; † Department of Genetics, Stanford University School of Medicine, Stanford, CA; ‡ Howard Hughes Medical Institute and Program in Epithelial Biology, Stanford University, Stanford, CA; § Department of Dermatology, Yale University School of Medicine, New Haven, CT; ** Yale Comprehensive Cancer Center, New Haven, CT; †† Department of Genetics, Yale University School of Medicine, New Haven, CT 06520-8005; ‡‡ Biostatics Resources, Keck Laboratory, Yale University, New Haven, CT 06520; §§ Department of Epidemiology and Public Health, Yale University School of Medicine, New Haven, CT 06520

**Keywords:** miR-150, miR-183, histopathology, skin cancer, expression profiling

## Abstract

Basal cell carcinomas (BCCs) are the most common cancers in the United States. The histologic appearance distinguishes several subtypes, each of which can have a different biologic behavior. In this study, global miRNA expression was quantified by high-throughput sequencing in nodular BCCs, a subtype that is slow growing, and infiltrative BCCs, aggressive tumors that extend through the dermis and invade structures such as cutaneous nerves. Principal components analysis correctly classified seven of eight infiltrative tumors on the basis of miRNA expression. The remaining tumor, on pathology review, contained a mixture of nodular and infiltrative elements. Nodular tumors did not cluster tightly, likely reflecting broader histopathologic diversity in this class, but trended toward forming a group separate from infiltrative BCCs. Quantitative polymerase chain reaction assays were developed for six of the miRNAs that showed significant differences between the BCC subtypes, and five of these six were validated in a replication set of four infiltrative and three nodular tumors. The expression level of miR-183, a miRNA that inhibits invasion and metastasis in several types of malignancies, was consistently lower in infiltrative than nodular tumors and could be one element underlying the difference in invasiveness. These results represent the first miRNA profiling study in BCCs and demonstrate that miRNA gene expression may be involved in tumor pathogenesis and particularly in determining the aggressiveness of these malignancies.

Nonmelanoma skin cancers, 80% of which are basal cell carcinomas (BCCs), are the most common cancers in the United States, accounting for approximately 3.5 million new diagnoses each year (Rogers *et al.* 2010). The incidence of this tumor type is increasing in many countries around the world (Gallagher *et al.* 1990; Hannuksela-Svahn *et al.* 1999; Karagas *et al.* 1999; Levi *et al.* 2001). BCC is a treatable cancer but can still be associated with significant morbidity. Most BCCs are located on the head and neck, and while rarely metastatic, these tumors can invade local tissues, and treatment can be disfiguring (Netscher *et al.* 2011).

There are several subtypes of BCCs, which may present with clinically diverse features. A common histopathologic classification system (Lang and Maize 1986; Sexton *et al.* 1990) divides the tumors into five main categories. The most common, nodular BCC, appears grossly as a translucent or pearly papule with telangiectasias coursing through it. Microscopically, nodular BCC is characterized by a compact mass of cells resembling the basal layer of the epidermis but extending into the dermis. These tumors have sharp margins with a palisaded peripheral border separating tumor from normal tissue. Micronodular BCCs are similar in gross appearance to nodular BCCs but microscopically are composed of many small tumor nodules rather than a single compact tumor mass. The superficial subtype consists of a flat erythematous plaque, which variably has scale, a translucent border, and areas of hypopigmentation, atrophy or scarring. Histology shows tumor nests budding from the epidermis. Infiltrating (also known as aggressive-growth) BCCs can have many different gross appearances but are characterized histologically as irregular islands of tumor cells with jagged projections into surrounding tissue. The morpheaform subtype of the aggressive growth category resembles a plaque of localized scleroderma, with indistinct borders. Microscopically, there is intense stromal proliferation and collagen production surrounding small irregular islands of tumor cells. The histopathologic subtype correlates with the risk of recurrence after surgical excision (Sexton *et al.* 1990). Nodular and superficial BCCs are relatively straightforward to extirpate. Infiltrative and morpheaform BCCs have unpredictable margins and typically are treated by the Mohs microscopically controlled technique, which ensures a high rate of cure. The same rationale applies to micronodular tumors, which have an intermediate risk of recurrence.

At least 10% of tumors contain elements of more than one subtype (Carr *et al.* 2007; Jones *et al.* 1998; Rippey 1998), and dermatopathologists often classify BCCs according to which histologic pattern is present in the bulk of the tumor. Generally the histology of a BCC does not change over time, although with recurrence, more aggressive BCC may be noted. (Boulinguez *et al.* 2004; Dixon *et al.* 1991; Lang and Maize 1986; Rippey 1998).

The relative stability in biologic behavior among subtypes may reflect somatic genetic or epigenetic alterations that can be stably transmitted from parent tumor cell to daughter cell. However, among the known genetic alterations in BCCs, none correlates with subtype. Activation of the hedgehog signal transduction pathway may be a necessary, if not sufficient, step in the development of BCC (Epstein 2008; Sidransky 1996). The hedgehog signal is received and transduced at the membrane via a receptor complex consisting of patched (PTCH), a negative regulator switched off by hedgehog binding, and smoothened, which activates the pathway when released from inhibition by PTCH. Mutation analysis of BCCs indicates that a high percentage have inactivating *PTCH* mutations (Bodak *et al.* 1999; Gailani *et al.* 1996a; Gailani *et al.* 1996b; Reifenberger *et al.* 2005). Almost all of those without *PTCH* mutations have activating mutations in *SMO* (Reifenberger *et al.* 2005; Xie *et al.* 1998). Minute BCCs are as likely as large tumors to have *PTCH* mutations. In addition all histologic subtypes, whether primary or recurrent, have a high frequency of loss of PTCH or activation of SMO. Neither of two other genes (*TP53* and *HRAS*) known to undergo mutation in BCCs correlates with tumor size, histology, or rate of recurrence (Gailani *et al.* 1996a).

Although an underlying molecular basis for differentiation into subtypes remains elusive, messenger RNA (mRNA) expression profiling has been applied to BCCs in an attempt to identify genes whose perturbation in expression may represent a proximate cause of the biologic features of these tumors. In some studies of mRNA authors identified genes whose expression was different in normal skin *vs.* basal cell tumors (Asplund *et al.* 2008; O’Driscoll *et al.* 2006), but the normal cell of origin with which BCC tissue should be compared is probably a hair follicle stem cell (Sellheyer 2011), and therefore these expression differences may not be specific to tumorigenesis. The ability to distinguish histopathologic subtypes was limited. Global mRNA expression patterns in superficial, nodular, and morpheaform BCCs failed to distinguish these tumor types in unbiased analysis (Yu *et al.* 2008). Likewise, a more limited study using a microarray with approximately 2000 genes failed to distinguished morpheaform from nodular BCCs in unbiased clustering (Howell *et al.* 2005).

MicroRNAs (miRNAs) are small, regulatory RNAs that average 22 bp in length (He and Hannon 2004; Huang *et al.* 2011; Lee *et al.* 2003) and whose misexpression is common in many forms of cancer and often correlates with prognosis, outcome, and subtype (Farazi *et al.* 2011; Lu *et al.* 2005; Martens-Uzunova *et al.* 2011; Meng *et al.* 2007; Schramedei *et al.* 2011; Shenouda and Alahari 2009; Youssef *et al.* 2011). Mature miRNAs are bound by the DICER complex and then directed to target mRNAs via a 6-bp seed sequence in their 5′ region (Lewis *et al.* 2005). Upon the binding the mRNA, they either target it for degradation or inhibit translation. miRNAs are grouped into families defined by homology to a common ancestor (Heimberg *et al.* 2008). Although members of the same family are often located adjacent to each other and co-expressed, there are examples of family members being located on multiple chromosomes, and neither similar expression nor even similar function are requirements for miRNAs to be grouped into a family. Approximately 1000 miRNAs have been characterized in humans, and new types continue to be discovered. miRNA expression has not previously been characterized in BCCs, leaving a potentially important facet of the differences between the subtypes unexplored.

In this study, global miRNA expression was quantified by high-throughput sequencing in a discovery set of eight nodular and eight infiltrative BCCs to determine whether the overall pattern of expression distinguishes these subtypes. Six miRNAs that showed significantly different expression in the two tumor classes were validated in a replication set of three nodular and four infiltrative samples. These results have important implications for understanding molecular mechanisms of BCC pathogenesis.

## Materials and Methods

### Acquisition of samples

Tumors were excised by Mohs surgery in which the central tumor mass was debulked and the margins excised under microscopic control. The debulked material was snap frozen in liquid nitrogen and then stored at −80°. This material was shown in previous studies of BCCs obtained from the same Mohs surgeon to contain minimal contaminating normal tissue (Gailani *et al.* 1996a, 1996b; Ziegler *et al.* 1993). A discovery set of eight nodular and eight infiltrative tumors was selected for sequencing (Table 1). Three nodular and four infiltrative tumors were used as a replication set for biological validation of select miRNAs . The study was approved by the Yale University School of Medicine Human Investigation Committee.

**Table 1:** Characteristics of basal cell carcinomas

Identifier	Subtype	Comments	Size, cm	Recurrent/Primary	Age/Gender	Sequencing	qPCR
Discovery							
Nod 1	Nodular		3.1 × 2.2	Primary	F/77	x	x
Nod 2	Nodular		2.1 × 1.6	Primary	F/82	x	
Nod 3	Nodular		1.1 × 0.3	Recurrent	M/68	x	
Nod 4	Nodular		1.6 × 1.5	Primary	M/67	x	x
Nod 5	Nodular		1.3 × 0.9	Primary	F/81	x	x
Nod 6	Nodular		2.0 × 1.0	Primary	M/64	x	x
Nod 7	Nodular		2.0 × 1.8	Primary	M/69	x	x
Nod 8	Nodular	Sebaceous differentiation	4.3 × 3.3	Primary	M/84	x	
Inf 1	Infiltrative		2.6 × 2.2	Recurrent	M/79	x	
Inf 2	Infiltrative	Nodular component	1.0 × 0.5	Primary	M/45	x	
Inf 3	Infiltrative		2.0 × 0.7	Primary	M/90	x	
Inf 4	Infiltrative		1.2 × 1.1	Primary	M/44	x	
Inf 5	Infiltrative		2.7 × 1.4	Primary	M/73	x	x
Inf 6	Infiltrative		1.3 × 1.2	Primary	M/74	x	x
Inf 7	Infiltrative	Morpheaform component	2.0 × 2.0	Primary	M/68	x	x
Inf 8	Infiltrative		2.2 × 0.7	Primary	M/69	x	x
Replication							
Nod 9	Nodular		1.8 × 1.7	Primary	M/78		x
Nod 10	Nodular		2.0 × 1.4	Primary	M/71		x
Nod 11	Nodular		1.3 × 1.1	Primary	M/42		x
Inf 9	Infiltrative		1.6 × 1.5	Primary	F/75		x
Inf 10	Infiltrative		2.8 × 2.0	Recurrent	M/49		x
Inf 11	Infiltrative		3.0 × 0.9	Primary	M/77		x
Inf 12	Infiltrative		2.0 × 1.4	Primary	F/41		x

qPCR, quantitative polymerase chain reaction.

### Preparation of RNA

Total RNA was extracted from tumors using the mirVana total RNA extraction kit (Ambion, Austin, TX). A total of 1 μg of total RNA was then prepared into libraries using the Illumina v1.5 small RNA kit (Illumina, San Diego, CA), which selects for miRNA and other DICER processed RNAs by binding selectively to 3′ hydroxyl groups resulting from cleavage by RNA-processing enzymes. Samples were then sequenced on the Illumina Genome Analyzer.

### Data analysis

miRExpress (Wang *et al.* 2009) was used to collapse raw fastq files to unique tags and number of appearances and to trim adapter sequences from each unique tag. Trimmed tag sequences were aligned to annotated miRNA precursor sequences in miRBase v15.0 (Griffiths-Jones 2010), and number of hits on each annotated miRNA was counted. Only reads that mapped to miRNA precursor sequences in this database were used for further analysis; all reads that failed to map to a known miRNA were discarded.

Global normalization of the miRNA expression profiles was performed using quantile normalization. The function “normalizeQuantiles” in R Bioconductor package limma (Smyth *et al.* 2005) is used without a reference distribution. First, the raw counts of all miRNAs in each sample were sorted separately. Then, the highest count of each sample was replaced by the mean of the highest counts of all samples, the second highest count was replaced by the mean of the second highest counts, and so on. The normalized miRNA expression profiles were used to detect differentially expressed miRNAs between nodular and infiltrative samples using edgeR (Robinson *et al.* 2010). To summarize in brief, a negative binomial model was used with estimated overdispersion (relative to the Poisson) from the miRNA expression profiles. The dispersion parameter of each miRNA was estimated by the tagwise dispersion. Then, an exact test was performed to test for differential expression between the two sample groups. The correction for multiple hypothesis testing was achieved by use of the Benjamini and Hochberg approach for controlling the false-discovery rate (Benjamini and Hochberg 1995). Highly expressed miRNAs were determined by simple rank order of normalized expression levels. The principal components analysis was performed on total miRNA expression using the princomp function in the standard R package, and the top two principal components accounting for 89.7% of total variance were plotted.

### Validation

Quantitative polymerase chain reaction (qPCR) assays were developed for eight differentially expressed miRNAs. The standard Taqman small RNA assay procedure (Applied Biosystems, Carlsbad, CA), with U18 serving as a control, was used for miRNA quantitation. Validation targets were selected to survey a range of expression levels from the eight miRNAs that had a *P*-value corrected for false discovery rate < 0.01 significance between nodular and infiltrative subtypes. Specifically, miRNAs were selected on the basis of *P*-value corrected for multiple comparisons, sufficient overall expression, and magnitude of difference between classes. Of these eight assays, six provided adequate results. Those with an average Ct value greater than 30 were discarded because of insufficient expression. Data were analyzed by ΔΔCt relative to mean expression for each sample group (Livak *et al.* 2001).

### Target prediction

Potential targets for each miRNA were determined using the intersection of lists from PicTar (Krek *et al.* 2005) and TargetScanS (Lewis *et al.* 2005). This combined dataset rather than predictions from literature was used for gene ontology analysis to avoid bias. To assess the enrichment of miRNA target genes in functional categories, the R package topGO (Alexa *et al.* 2006) was used against all Gene Ontology (Harris *et al.* 2004) biological process terms. Specifically, the Fisher’s exact test was applied for testing the overlap between the targets of each of 12 selected miRNAs and each GO term, controlling for the ~18,000 total human miRNA target genes used in the TargetScan database (Lewis *et al.* 2005).

## Results

### miRNA RNA sequencing of infiltrative and nodular tumors reveals highly expressed miRNAs

To investigate the differences between infiltrative and nodular BCCs, total RNA was extracted from eight infiltrative and eight nodular BCCs and small RNAs were sequenced on the Illumina Genome Analyzer using the Illumina siRNA v1.5 kit. This kit selects for miRNA and ncRNAs as the result of an adapter that preferentially ligates to the 3′ hydroxyl resulting from DICER processing. A total of 221,233,365 reads (121,428,390 nodular, 99,805,205 infiltrative; supporting information, Table S1) was obtained with a range of 7,177840 to 24,914,601 reads for each sample. Of these, 179,510,825 (21,628,080 to 3,074,442) passed quality filters on the basis of successful base calls. After removal of adapter reads and sequencing artifacts, 134,778,713 reads mapped to the human genome. These reads ranged in length from 15 bp to 35 bp, with peaks at 22 bp and 34 bp (Figure S1). The peak at 22 bp was composed primarily of known mature miRNAs, whereas the peak from 29 to 35 bp was mainly transfer RNA fragments but also contained ncRNAs, partial precursor miRNAs, and degraded products from ribosomal and mRNAs. Processed transfer RNAs also have a compatible 3′ hydroxyl end and cause the vast majority of non-miRNA contamination as the result of their abundance. Of the postfilter reads, 57,098,138 (42.4%) mapped to validated human miRNAs. Of these, 59.6% were from nodular samples whereas 40.4% were from infiltrative samples.

The 57,098,138 miRNA sequences mapped to 952 annotated human miRNAs (miRbase v15.0). Of these, 18 accounted for the majority of all reads and each of the 18 represented more than 1% of the total reads (Figure S2). A total of 74 miRNAs accounted for 95% of all miRNA expression, and 162 accounted for 99% of all expression after normalization. The remaining 1% of reads came from 790 different miRNAs. A total of 476 miRNAs had an average of less than 10 normalized reads per sample, indicating that expression was very low or nonexistent. The most highly expressed miRNA (12% of expression) was miR-21, a known oncogene that represses a variety of tumor suppressors such as *PTEN* and *PCDC4*. Other highly expressed miRNAs included the Let-7 family, which is involved in regulating cell proliferation, as well as miR-143, miR-182, miR-148a, and miR-378.

### Differentially expressed miRNAs distinguish infiltrative and nodular tumors

**Figure 1:**
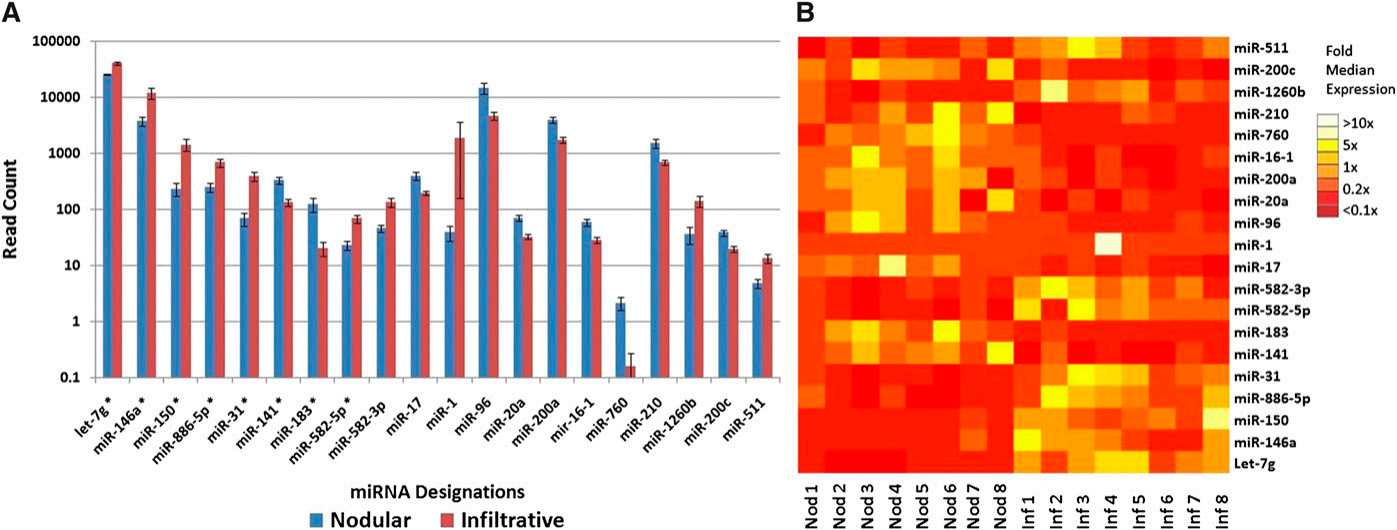
Twenty miRNAs had statistically significant differences in expression (false discovery rate <0.01) between nodular and infiltrative BCCs. (A) A sample representing a range of expression, indicated by an asterisk, was chosen for validation by qPCR. (B) A heatmap of differentially expressed miRNAs shows that although miRNA expression patterns tend to be consistent within a given class, many tumors show anomalous expression for a handful of miRNAs.

In addition to determining the rank order of highly expressed miRNAs, we also examined expression differences between nodular and infiltrative tumors. When we used the method described previously (in *Materials and Methods*), 20 mature miRNAs showed differential expression at *P* = 0.01 after Benjamani correction (Figure 1, A and B).

**Figure 2:**
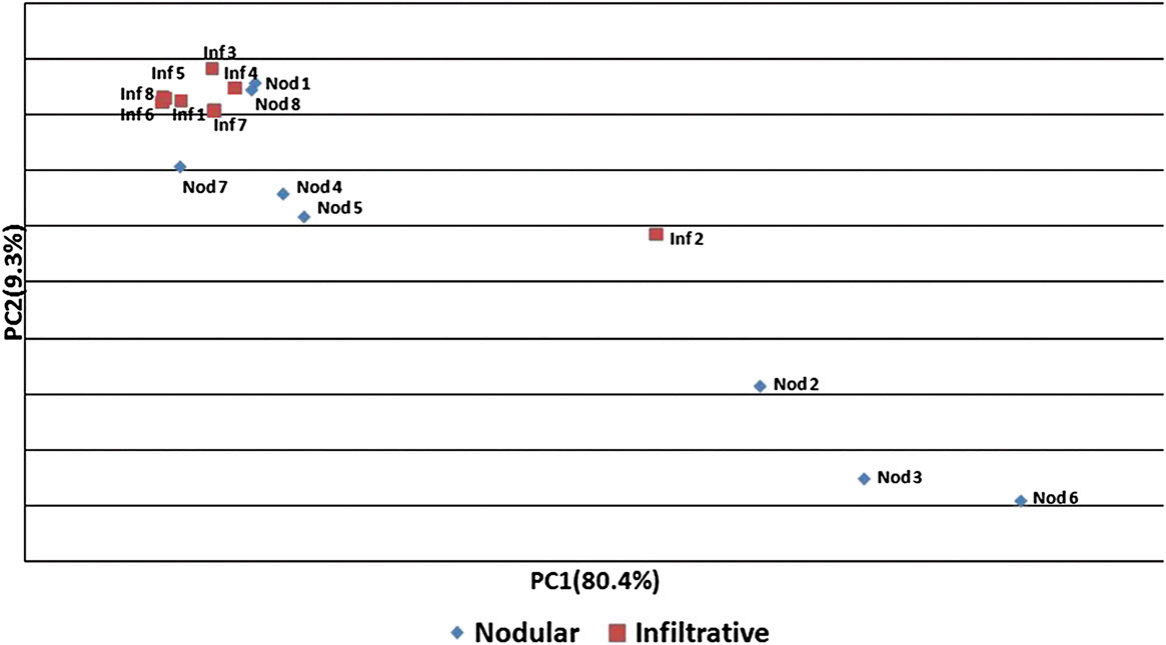
Principal components analysis of 16 tumors on the basis of total miRNA expression. Infiltrative tumors clustered tightly with the exception of Inf 2, which was the smallest of the infiltrative tumors and which had a significant nodular component. Nodular tumors had a much broader distribution, reflecting the increased heterogeneity of the subtype. Nod1 and Nod8, which clustered near the infiltrative tumors, were the largest of the nodular class. The axes, principal components one and two, are produced by determining the variability of miRNA expression within the total dataset and collapsing correlating variance into a reduced set of values. The first two components account for 89.7% of the total variance within the dataset; none of the remaining components account for more than 3.0%.

To evaluate the ability of the miRNA expression profile to distinguish between subtypes, a principal components analysis was used to cluster samples in an unbiased fashion (Figure 2). Infiltrative samples clustered closely with the exception of tumor Inf2. On pathologic review of all tumors in the discovery set, the Inf 2 infiltrative BCC sample was the smallest in its class and was found to have a significant element with nodular histology, indicating that it may be less aggressive. Nodular tumors had a broad distribution, which reflected the broader spectrum of tumors falling under the nodular classification. Nod 1 and Nod 8, which clustered close to the infiltrative tumors, were the largest in their class, perhaps indicating more aggressive biologic behavior. However, when we factored all infiltrative and nodular tumors together, we found that there was very little correlation between tumor area (product of two largest dimensions) and either of the two major principal components (*R*^2^ = 0.095), indicating that clustering was not related to tumor size but rather to histologic type. In addition, we found no correlation between patient age and expression (*R*^2^ = −0.042) or patient gender and expression (*R*^2^ = −0.008) with gender as a binomial function.

**Figure 3:**
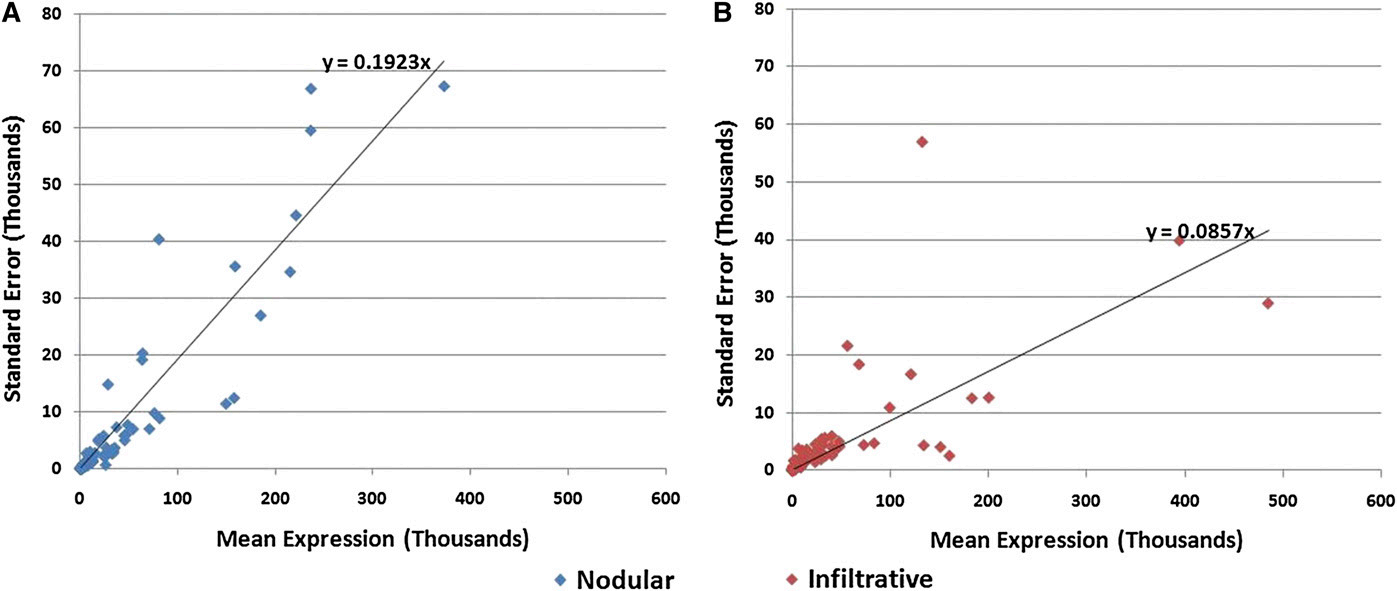
Characterization of variance within BCC classes. Standard error *vs.* mean expression was graphed for each miRNA. Nodular tumors (A) have a standard error relative to the mean of 0.1923, approximately twice that of infiltrative specimens (B) at 0.0857. These data suggest that the wide distribution of nodular tumors in principal component analysis is attributable to an overall pattern of higher variance in expression of miRNAs rather than a few highly variable miRNAs.

To obtain better insight into tumor heterogeneity, we quantified overall variance within each class by plotting the standard deviation *vs.* mean expression of each miRNA for nodular (Figure 3A) and infiltrative (Figure 3B) tumors. On the basis of the best-fit line, nodular tumors (slope = 0.1923) showed twice as much variation in expression as infiltrative (slope = 0.0857). To determine whether the greater variance in nodular miRNA expression was attributable to a few outliers or an overall pattern of greater variance in expression, we identified the greatest outliers by using a normal q-q plot and removed them from the analysis. Although the results of the regression plot shifted slightly (slope for nodular, 0.1798; slope for infiltrative, 0.0918), their overall relationship remained the same.

### Validation of differentially expressed miRNAs

**Figure 4:**
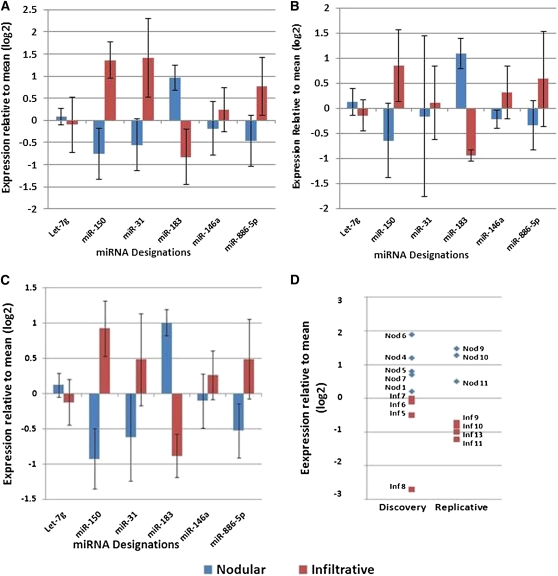
Validation of differentially expressed miRNAs by qPCR. Six miRNAs were assessed by Taqman assays. Assays for miR-141 and miR-582-5p failed because of low expression levels (see *Materials and Methods*). A) Among nine tumors from the discovery set in which adequate RNA was available, qPCR mirrored RNAseq in the direction and approximate magnitude of differences between the two tumor types except for Let-7g. (B) A replication set of seven tumors showed concordance in direction with qPCR results in the discovery set. (C) Upon combining the analytical and biological validation sets, three of six miRNAs, miR-150, 183, and 886-5p, were significantly different (*P* < 0.05) and two others, miR-31 and 146, trended in the same direction as shown by RNA sequencing but failed to reach significance. (D) Expression of miR-183 showed no overlap in nodular tumors compared with infiltrative tumors in both the discovery and replication set. Y-axis values are provided in log(2) such that a value of one indicates a twofold difference between samples.

To confirm that miRNA abundance determined by sequencing was an accurate reflection of expression levels, a second quantitative method, qPCR, was used to measure levels of six miRNAs in nine of 16 of the original sequenced tumors (Figure 4A). The remaining seven tumors had insufficient RNA remaining for Taqman assays. For five out of six miRNAs (miR-150, miR-31, miR-183, miR-146a, and miR-886-5p), expression differences between tumor classes were concordant with sequencing. Let 7g, which showed a small but significant increase in expression in infiltrative tumors as determined by sequencing, showed a small but opposite trend in the Taqman assay.

When, the expression of the six miRNAs was examined in a replicate set consisting of four infiltrative and three nodular tumors (Figure 4B), differences between the two BCC subtypes were concordant with qPCR in the discovery set. Combining Taqman results from the discovery and replication sets (Figure 4C), five miRNAs remained concordant with sequencing results. As the result of variability in expression among tumors, only three of these differences were statistically significant (*P* < 0.05). Of these, miR-183 was able to distinguish between tumor subtypes with no overlap between the two classes (Figure 4D).

### Predicted targets of miR-183 suggest a role in cell motility

Both PicTar and TargetScanS predicted a combined set of 125 genes to be targets of miR-183. GO classification of genes regulated by miR-183 showed a significant (*P* < 0.01) enrichment in sixty-one categories (Table S2). Fifteen of these categories, including five of the top 10 most significant, were involved in actin polymerization. Eleven were involved in morphogenesis and differentiation, and three categories were involved in apoptosis.

## Discussion

BCCs can be divided into several subtypes on the basis of histopathologic features and biologic behavior. The molecular basis for the differentiation of these tumors into different classes is not established. Virtually all BCCs have underlying *PTCH* or *SMO* mutations leading to activation of the hedgehog pathway (Bodak *et al.* 1999; Gailani *et al.* 1996a, 1996b; Reifenberger *et al.* 2005; Xie *et al.* 1998). Additional mutations in other genes—particularly *TP53* (Ziegler *et al.* 1993)— are fairly common but do not correlate with clinical characteristics of the tumors (Gailani *et al.* 1996a). Other, as-yet undiscovered, genetic or epigenetic mechanisms must play a role in the specification of subtype.

The current study examined global miRNA expression as it relates to the most common benign and aggressive subtypes, that is, nodular and infiltrative, respectively. miRNA expression was shown to correlate with subtype through unbiased clustering methods. One outcome of the study was that nodular tumors tended to show more variation in miRNA expression than infiltrative tumors. Nodular BCCs are the most common type, and tumors not clearly falling into other categories are classified as nodular or “no special type” (Carr *et al.* 2007). Among nodular tumors, there are rare distinctive patterns of morphology such as pseudo-glandular, cystic, and clear cell (Carr *et al.* 2007; Saldanha *et al.* 2003) although most are not otherwise differentiated. The broad clustering of this tumor type on principal components analysis may reflect diversity in the biology of nodular BCCs.

Many of the miRNAs that helped distinguish the two classes were previously identified in studies of cancer. Expression of one specific miRNA, miR-183, showed no overlap between the classes. A known role of the miR-183 family in normal tissue is regulation of neurosensory cell specification and maintenance of normal basal-apical gradients in maturing cochlear hair cells. Although miR-183 is not known to play a role in skin development, its role in neurosensory stem cell differentiation may be mirrored in skin cells. In breast and lung cancer cell lines, miR-183 overexpression has been shown to inhibit cell migration and invasive behavior *in vitro* (Lowery *et al.* 2010; Wang *et al.* 2008), and dysregulation of expression has been observed in primary tissue samples from colon, bladder, breast, and medullary thyroid carcinoma (Abraham *et al.* 2011; Bandres *et al.* 2006; Han *et al.* 2011; Lowery *et al.* 2010). In addition, gene ontology analysis of predicted miR-183 targets revealed many genes involved in actin polymerization and cell projection in our study and others (Wang *et al.* 2008), and failure to down-regulate such genes may relate to increased cell motility and invasiveness. The *in vitro* studies and gene ontology studies of miR-183 targets suggest that infiltrative BCCs, with relatively low expression of mir-183, would be expected to have a greater tendency to invade.

In addition to miR-183, many other miRNAs and miRNA families with previously described roles in cancer development and progression show expression differences between the two classes. miR-17 and miR-20a are members of the same family and are expressed approximately twofold greater in nodular *vs.* infiltrative carcinomas. They have been found to be down-regulated in head and neck squamous cell carcinomas (Hui *et al.* 2010). The miR-141, 200a, and 200c are also members of the same family and are expressed approximately twofold greater in nodular carcinomas *vs.* infiltrative. They are believed to be regulated by C-MYC, and may be involved in the WNT and beta-catenin signaling pathways (Saydam *et al.* 2009). In addition, the miR-141/200a/200c family has been found to be frequently dysregulated in squamous cell carcinomas and melanomas, especially metastatic melanomas (Elson-Schwab *et al.* 2010; Imanaka *et al.* 2011). In addition, their down-regulation has been linked to increased invasiveness in melanomas and nasopharyngeal carcinomas(Xia *et al.* 2010). This is believed to be at least partially attributable to their down-regulation of *ZEB1* and *ZEB2*, which inhibit E-caderin (Saydam *et al.* 2009; Wu *et al.* 2011; Xia *et al.* 2010). Other miRNAs profiled included miR-210, which is expressed approximately twofold greater in nodular *vs.* infiltrative tumors and causes reduced progression in esophageal squamous cell carcinomas at least in part by down-regulating fibroblast growth factor receptor like-1 (*FGFRL-1*) (Tsuchiya *et al.* 2011). Again, the lower levels of these miRNAs in infiltrative tumors are consistent with their aggressive behavior.

Alterations in miRNA expression may be an important proximate cause, if not underlying cause, of the differentiation of BCCs into different histologic subtypes. Because miRNAs regulate a large number of genes, it can be challenging to attribute a specific tumor phenotype to over- or underexpression of any particular miRNA target. However, broad miRNA expression patterns have been linked to clinical outcomes, and this study supports miRNA profiling as a means of classifying and predicting the behavior of BCCs (Farazi *et al.* 2011).

## Supplementary Material

Supporting Information
