# Supplementary material for: Correlation of Global MicroRNA Expression With Basal Cell Carcinoma Subtype
Source: G3 (Bethesda). 2012 Feb 1;2(2):279–86. doi: 10.1534/g3.111.001115 (PMC3284335; doi:10.1534/g3.111.001115)
Supplement: Supporting Information [file supp_2_2_279__index.html]

Supporting Information 

# Correlation of Global MicroRNA Expression With Basal Cell Carcinoma Subtype

## Supporting Information for Heffelfinger *et al.*, 2012

**Files in this Data Supplement:**

- Supporting Information - Figures S1 and S2 and Tables S1 and S2 (PDF, 359 KB)
- Figure S1 - Distribution of sequencing reads for all tumor samples (PDF, 125 KB)
- Figure S2 - Highly expressed miRNAs in basal cell carcinomas (PDF, 106 KB)
- Table S1 - Sequenced BCC Read Statistics (PDF, 41 KB)
- Table S2 - GO categories of genes regulated by miR-183 (PDF, 115 KB)
