# Supplementary material for: Correlation of Global MicroRNA Expression With Basal Cell Carcinoma Subtype
Source: G3 (Bethesda). 2012 Feb 1;2(2):279–86. doi: 10.1534/g3.111.001115 (PMC3284335; doi:10.1534/g3.111.001115)
Supplement: Supporting Information [file supp_2.2.279_001115SI.pdf]

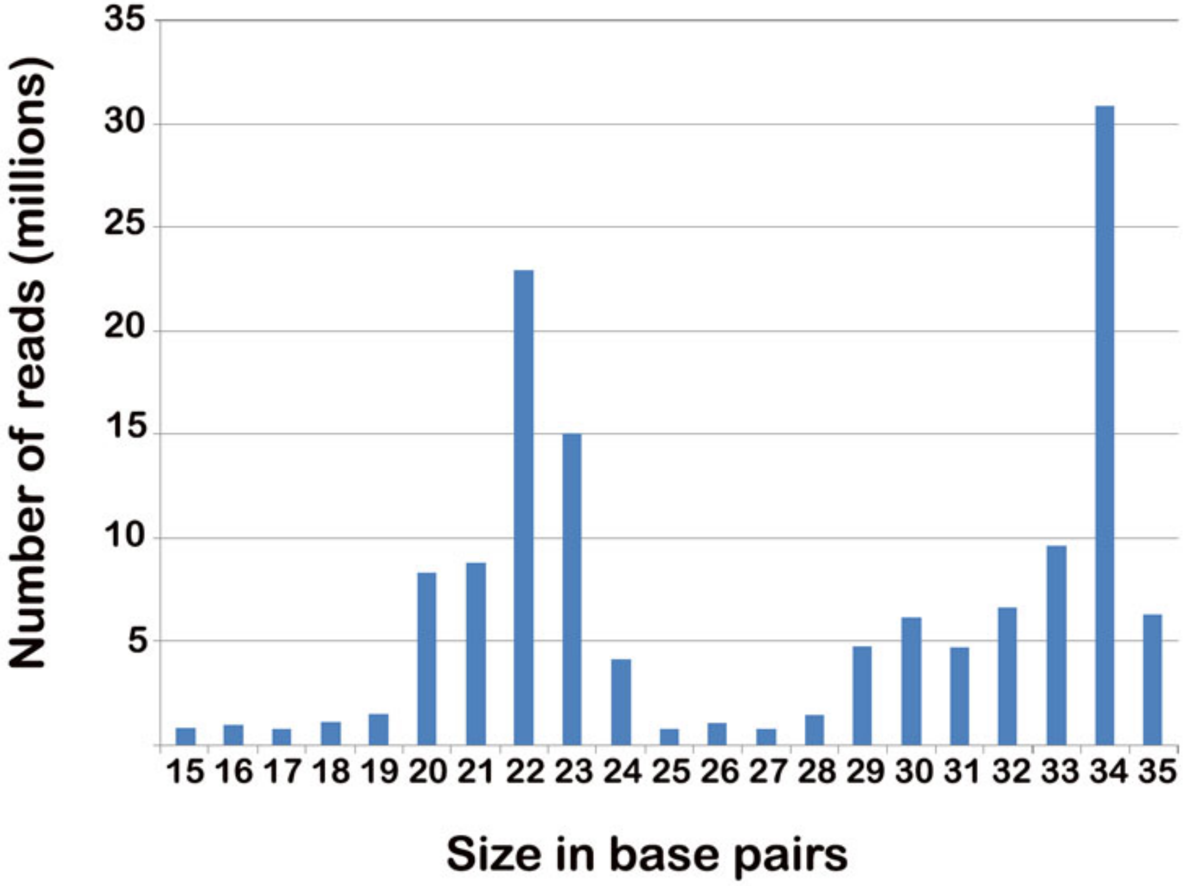

Figure S1 Distribution of sequencing reads for all tumor samples. All reads that mapped to the human genome with two mismatches or fewer were counted and graphed based on length. The peak centered on 22bp consists primarily of reads from mature miRNAs. Reads mapping to the 34bp are primarily from tRNA fragments, which are detected by this sequencing method due to their enzymatic processing which leaves ends similar to miRNA. The 34bp peak also contains ncRNAs, snRNAs, precursor miRNAs, and degraded mRNA and rRNA fragments.

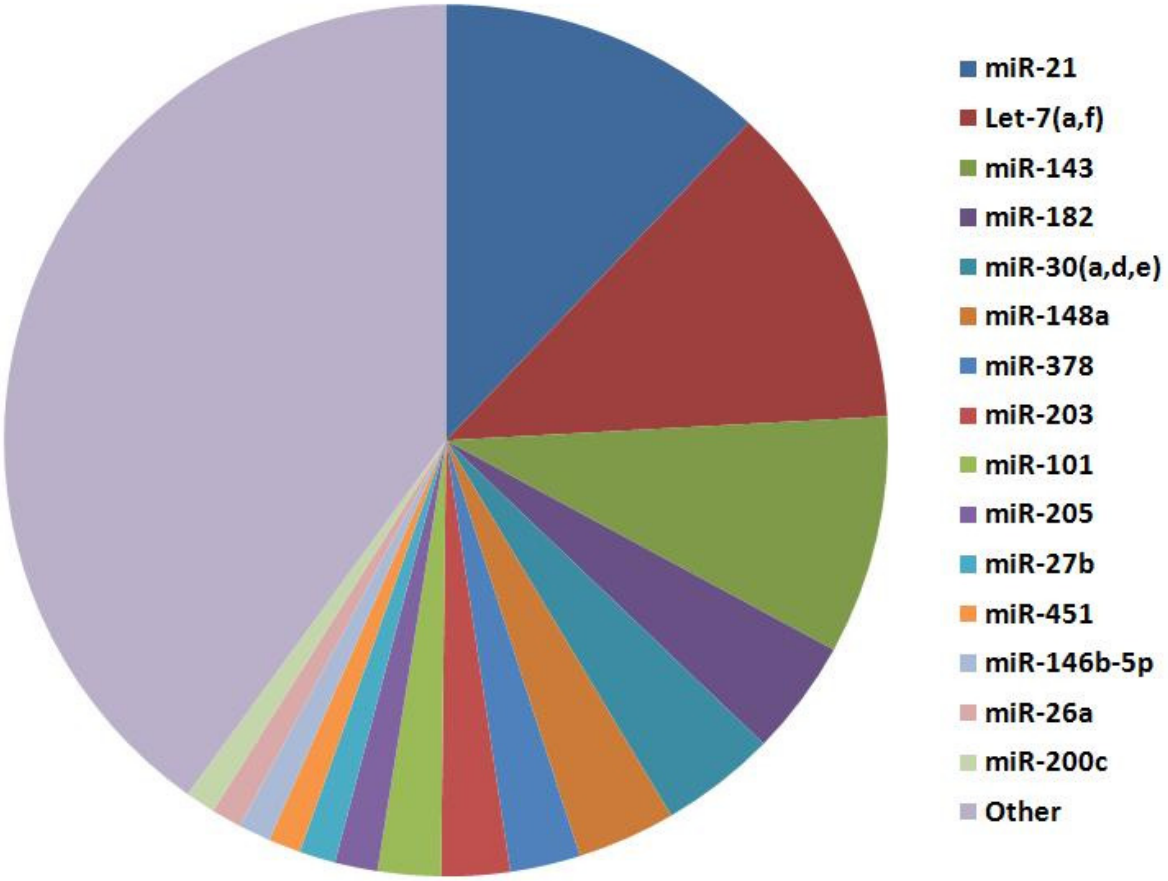

Figure S2 Highly expressed miRNAs in basal cell carcinomas. miR-21, which accounts for twelve percent of all miRNA expression, is a known oncogene repressing a variety of tumor suppressors such as PTEN and PCDC4. The second most highly expressed miRNAs at eleven percent are the Let-7 family, the majority of whose expression is accounted for by Let-7a. 934 miRNAs, each representing less than one percent of total expression, were grouped into the category, “other”.

Table S1 Sequenced BCC Read Statistics

	Lane 1	Lane 2	Total Reads	Reads Passing Quality Filter*	Contamination (ie adapters)	No match	Post filter mapped	Reads mapped to known miRNAs
Nod 1	8,004,773	8,536,662	16,541,435	13,877,215	2,344,855	802,777	10,729,583	4,352,584
Nod 2	7,971,342	8,927,821	16,899,163	14,602,747	5,191,154	988,352	8,423,241	5,812,998
Nod 3	7,596,811	11,067,243	18,664,054	17,399,390	2,956,014	490,981	13,952,395	3,060,949
Nod 4	4,221,903	6,699,042	10,920,945	9,761,882	2,462,482	1,202,086	6,097,314	3,765,975
Nod 5	13,589,899		13,589,899	12,511,028	541744	500,486	11,468,798	1,896,552
Nod 6	11,350,436	12,437,593	23,788,029	18,085,699	2,084,562	1,840,380	14,160,757	8,935,077
Nod 7	3,868,235	5,805,924	9,674,159	6,470,845	592,749	431,877	5,446,219	3,376,554
Nod 8	4,323,916	7,026,830	11,350,746	8,854,620	1,237,293	653,873	6,963,454	2,802,179
Inf 1	11,244,013	13,670,588	24,914,601	21,628,080	3880714	714156	17,033,210	6,376,847
Inf 2	4,359,210	6,582,290	10,941,500	9,682,543	2,208,253	194,324	7,279,966	914,638
Inf 3	5,058,043	7,307,966	12,366,009	10,069,215	4,228,105	488,188	5,352,922	2,131,473
Inf 4	4,907,060	7,010,204	11,917,264	9,598,784	2,488,425	1,074,571	6,035,788	3,116,381
Inf 5	4,756,008	6,803,272	11,559,280	8,569,815	801,131	758,693	7,009,991	3,162,598
Inf 6	4,476,827	6,783,141	11,259,968	8,349,506	1,073,296	425,232	6,850,978	3,586,415
Inf 7	4,119,433	5,549,310	9,668,743	6,975,014	619,914	434,036	5,921,064	3,094,521
Inf 8	7,177,840		7,177,840	3,074,442	534,580	486,829	2,053,033	712,397
Totals:			221,233,635	179,510,825	33,245,271	11,486,841	134,778,713	57,098,138

*Filter removes reads with excessive numbers of failed or ambiguous base calls.

Table S2. GO categories of genes regulated by miR-183.

GO.ID	Term	Number of genes in the genome in category	Number of miR-183 target genes category	Fold enrichment of genes category vs. expected value	P-value
GO:0042989	sequestering of actin monomers	4	3	100	2.40E-06
GO:0030837	negative regulation of actin filament polymerization	28	4	16.7	8.50E-05
GO:0032272	negative regulation of protein polymerization	30	4	16	0.00011
GO:0008064	regulation of actin polymerization or depolymerization	57	5	10.4	0.00012
GO:0006470	protein amino acid dephosphorylation	131	7	6.3	0.00012
GO:0030832	regulation of actin filament length	59	5	10	0.00014
GO:0031333	negative regulation of protein complex assembly	35	4	13.3	0.00021
GO:0044087	regulation of cellular component biogenesis	150	7	5.5	0.00028
GO:0007409	Axonogenesis	209	8	4.5	0.0004
GO:0008154	actin polymerization or depolymerization	74	5	7.9	0.0004
GO:0016311	Dephosphorylation	164	7	5	0.00048
GO:0043407	negative regulation of MAP kinase activity	45	4	10.5	0.00056
GO:0048667	cell morphogenesis involved in neuron differentiation	230	8	4.1	0.00075
GO:0030833	regulation of actin filament polymerization	51	4	9.3	0.0009
GO:0048812	neuron projection morphogenesis	237	8	4	0.00091

GO:0032956	regulation of actin cytoskeleton organization	89	5	6.7	0.00094
GO:0007015	actin filament organization	135	6	5.3	0.001
GO:0032970	regulation of actin filament-based process	93	5	6.3	0.00115
GO:0007041	lysosomal transport	25	3	14.3	0.00119
GO:0051494	negative regulation of cytoskeleton organization	57	4	8.3	0.00137
GO:0043508	negative regulation of JUN kinase activity	7	2	33.3	0.00145
GO:0030041	actin filament polymerization	60	4	7.8	0.00165
GO:0032271	regulation of protein polymerization	61	4	7.7	0.00176
GO:0007034	vacuolar transport	29	3	12	0.00184
GO:0030036	actin cytoskeleton organization	269	8	3.5	0.00203
GO:0045185	maintenance of protein location	64	4	7.4	0.0021
GO:0048858	cell projection morphogenesis	271	8	3.5	0.00212
GO:0042981	regulation of apoptosis	860	16	2.2	0.00234
GO:0007413	axonal fasciculation	9	2	25	0.00247
GO:0043067	regulation of programmed cell death	866	16	2.2	0.0025
GO:0010941	regulation of cell death	873	16	2.2	0.00271
GO:0032990	cell part morphogenesis	284	8	3.3	0.00284
GO:0030029	actin filament-based process	286	8	3.3	0.00296
GO:0000904	cell morphogenesis involved in differentiation	290	8	3.3	0.00322
GO:0000165	MAPKKK cascade	294	8	3.2	0.0035

GO:0009968	negative regulation of signal transduction	120	5	4.9	0.00351
GO:0023057	negative regulation of signaling process	122	5	4.9	0.00377
GO:0031098	stress-activated protein kinase signaling cascade	122	5	4.9	0.00377
GO:0031175	neuron projection development	307	8	3.1	0.00454
GO:0021955	central nervous system neuron axonogenesis	13	2	18.2	0.00523
GO:0001933	negative regulation of protein amino acid phosphorylation	42	3	8.3	0.00535
GO:0051493	regulation of cytoskeleton organization	134	5	4.4	0.00561
GO:0008333	endosome to lysosome transport	14	2	16.7	0.00606
GO:0070303	negative regulation of stress-activated protein kinase signaling cascade	14	2	16.7	0.00606
GO:0010648	negative regulation of cell communication	324	8	2.9	0.00625
GO:0030326	embryonic limb morphogenesis	87	4	5.4	0.00634
GO:0035113	embryonic appendage morphogenesis	87	4	5.4	0.00634
GO:0035467	negative regulation of signaling pathway	259	7	3.2	0.00646
GO:0043254	regulation of protein complex assembly	88	4	5.3	0.0066
GO:0006917	induction of apoptosis	329	8	2.9	0.00683
GO:0012502	induction of programmed cell death	330	8	2.9	0.00695
GO:0070302	regulation of stress-activated protein kinase signaling cascade	90	4	5.3	0.00714
GO:0043506	regulation of JUN kinase activity	47	3	7.5	0.00733
GO:0051128	regulation of cellular component organization	634	12	2.2	0.00734
GO:0032535	regulation of cellular component size	334	8	2.8	0.00746

GO:0015782	CMP-sialic acid transport	1	1	100	0.00848
GO:0035408	histone H3-T6 phosphorylation	1	1	100	0.00848
GO:0035107	appendage morphogenesis	96	4	4.9	0.00893
GO:0035108	limb morphogenesis	96	4	4.9	0.00893
GO:0010639	negative regulation of organelle organization	97	4	4.9	0.00926
GO:0051258	protein polymerization	99	4	4.8	0.00993
