# Supplementary material for: Correlation of Global MicroRNA Expression With Basal Cell Carcinoma Subtype
Source: G3 (Bethesda). 2012 Feb 1;2(2):279–86. doi: 10.1534/g3.111.001115 (PMC3284335; doi:10.1534/g3.111.001115)
Supplement: Supporting Information [file supp_2.2.279_FigureS1.pdf]

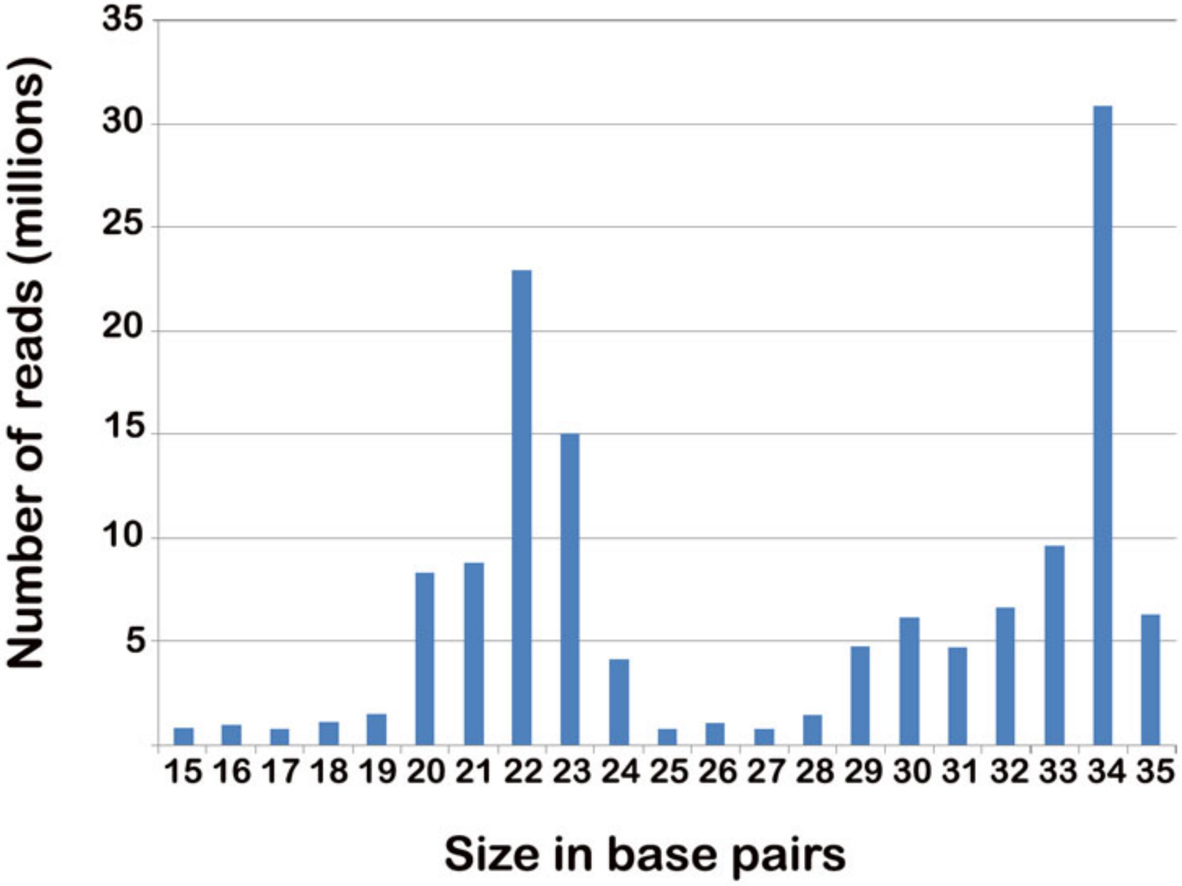


**Figure S1** Distribution of sequencing reads for all tumor samples. All reads that mapped to the human genome with two mismatches or fewer were counted and graphed based on length. The peak centered on 22bp consists primarily of reads from mature miRNAs. Reads mapping to the 34bp are primarily from tRNA fragments, which are detected by this sequencing method due to their enzymatic processing which leaves ends similar to miRNA. The 34bp peak also contains ncRNAs, snRNAs, precursor miRNAs, and degraded mRNA and rRNA fragments.
