# Supplementary material for: Correlation of Global MicroRNA Expression With Basal Cell Carcinoma Subtype
Source: G3 (Bethesda). 2012 Feb 1;2(2):279–86. doi: 10.1534/g3.111.001115 (PMC3284335; doi:10.1534/g3.111.001115)
Supplement: Supporting Information [file supp_2.2.279_FigureS2.pdf]

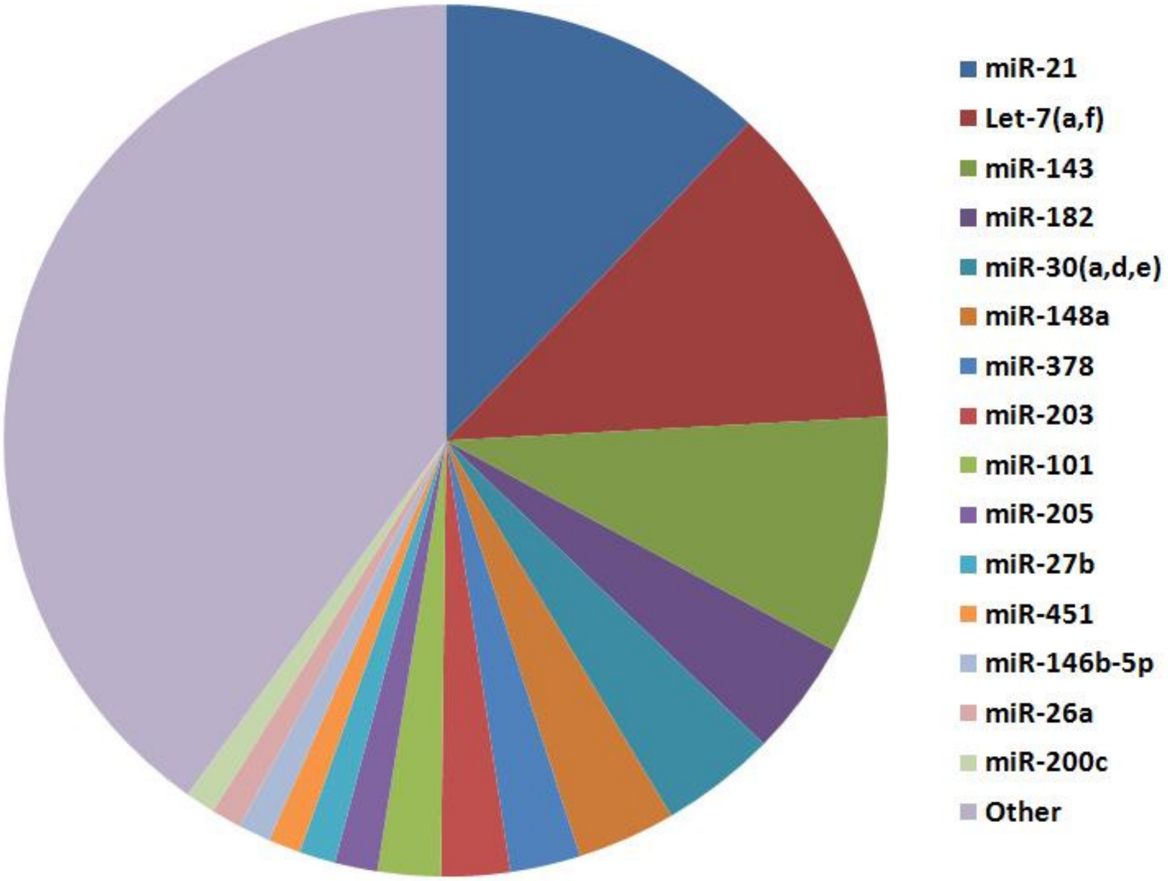

Figure S2 Highly expressed miRNAs in basal cell carcinomas. miR-21, which accounts for twelve percent of all miRNA expression, is a known oncogene repressing a variety of tumor suppressors such as PTEN and PCDC4. The second most highly expressed miRNAs at eleven percent are the Let-7 family, the majority of whose expression is accounted for by Let-7a. 934 miRNAs, each representing less than one percent of total expression, were grouped into the category, "other".
