# Supplementary material for: Correlation of Global MicroRNA Expression With Basal Cell Carcinoma Subtype
Source: G3 (Bethesda). 2012 Feb 1;2(2):279–86. doi: 10.1534/g3.111.001115 (PMC3284335; doi:10.1534/g3.111.001115)
Supplement: Supporting Information [file supp_2.2.279_TableS1.pdf]

Table S1 Sequenced BCC Read Statistics

	Lane 1	Lane 2	Total Reads	Reads Passing Quality Filter*	Contamination (ie adapters)	No match	Post filter mapped	Reads mapped to known miRNAs
Nod 1	8,004,773	8,536,662	16,541,435	13,877,215	2,344,855	802,777	10,729,583	4,352,584
Nod 2	7,971,342	8,927,821	16,899,163	14,602,747	5,191,154	988,352	8,423,241	5,812,998
Nod 3	7,596,811	11,067,243	18,664,054	17,399,390	2,956,014	490,981	13,952,395	3,060,949
Nod 4	4,221,903	6,699,042	10,920,945	9,761,882	2,462,482	1,202,086	6,097,314	3,765,975
Nod 5	13,589,899		13,589,899	12,511,028	541744	500,486	11,468,798	1,896,552
Nod 6	11,350,436	12,437,593	23,788,029	18,085,699	2,084,562	1,840,380	14,160,757	8,935,077
Nod 7	3,868,235	5,805,924	9,674,159	6,470,845	592,749	431,877	5,446,219	3,376,554
Nod 8	4,323,916	7,026,830	11,350,746	8,854,620	1,237,293	653,873	6,963,454	2,802,179
Inf 1	11,244,013	13,670,588	24,914,601	21,628,080	3880714	714156	17,033,210	6,376,847
Inf 2	4,359,210	6,582,290	10,941,500	9,682,543	2,208,253	194,324	7,279,966	914,638
Inf 3	5,058,043	7,307,966	12,366,009	10,069,215	4,228,105	488,188	5,352,922	2,131,473
Inf 4	4,907,060	7,010,204	11,917,264	9,598,784	2,488,425	1,074,571	6,035,788	3,116,381
Inf 5	4,756,008	6,803,272	11,559,280	8,569,815	801,131	758,693	7,009,991	3,162,598
Inf 6	4,476,827	6,783,141	11,259,968	8,349,506	1,073,296	425,232	6,850,978	3,586,415
Inf 7	4,119,433	5,549,310	9,668,743	6,975,014	619,914	434,036	5,921,064	3,094,521
Inf 8	7,177,840		7,177,840	3,074,442	534,580	486,829	2,053,033	712,397
Totals:			221,233,635	179,510,825	33,245,271	11,486,841	134,778,713	57,098,138

*Filter removes reads with excessive numbers of failed or ambiguous base calls.
