# Supplementary material for: Correlation of Global MicroRNA Expression With Basal Cell Carcinoma Subtype
Source: G3 (Bethesda). 2012 Feb 1;2(2):279–86. doi: 10.1534/g3.111.001115 (PMC3284335; doi:10.1534/g3.111.001115)
Supplement: Supporting Information [file supp_2.2.279_TableS2.pdf]

Table S2. GO categories of genes regulated by miR-183.

GO.ID	Term	Number of genes in the genome in category	Number of miR-183 target genes category	Fold enrichment of genes category vs. expected value	P-value
GO:0042989	sequestering of actin monomers	4	3	100	2.40E-06
GO:0030837	negative regulation of actin filament polymerization	28	4	16.7	8.50E-05
GO:0032272	negative regulation of protein polymerization	30	4	16	0.00011
GO:0008064	regulation of actin polymerization or depolymerization	57	5	10.4	0.00012
GO:0006470	protein amino acid dephosphorylation	131	7	6.3	0.00012
GO:0030832	regulation of actin filament length	59	5	10	0.00014
GO:0031333	negative regulation of protein complex assembly	35	4	13.3	0.00021
GO:0044087	regulation of cellular component biogenesis	150	7	5.5	0.00028
GO:0007409	Axonogenesis	209	8	4.5	0.0004
GO:0008154	actin polymerization or depolymerization	74	5	7.9	0.0004
GO:0016311	Dephosphorylation	164	7	5	0.00048
GO:0043407	negative regulation of MAP kinase activity	45	4	10.5	0.00056
GO:0048667	cell morphogenesis involved in neuron differentiation	230	8	4.1	0.00075
GO:0030833	regulation of actin filament polymerization	51	4	9.3	0.0009
GO:0048812	neuron projection morphogenesis	237	8	4	0.00091

GO:0032956	regulation of actin cytoskeleton organization	89	5	6.7	0.00094
GO:0007015	actin filament organization	135	6	5.3	0.001
GO:0032970	regulation of actin filament-based process	93	5	6.3	0.00115
GO:0007041	lysosomal transport	25	3	14.3	0.00119
GO:0051494	negative regulation of cytoskeleton organization	57	4	8.3	0.00137
GO:0043508	negative regulation of JUN kinase activity	7	2	33.3	0.00145
GO:0030041	actin filament polymerization	60	4	7.8	0.00165
GO:0032271	regulation of protein polymerization	61	4	7.7	0.00176
GO:0007034	vacuolar transport	29	3	12	0.00184
GO:0030036	actin cytoskeleton organization	269	8	3.5	0.00203
GO:0045185	maintenance of protein location	64	4	7.4	0.0021
GO:0048858	cell projection morphogenesis	271	8	3.5	0.00212
GO:0042981	regulation of apoptosis	860	16	2.2	0.00234
GO:0007413	axonal fasciculation	9	2	25	0.00247
GO:0043067	regulation of programmed cell death	866	16	2.2	0.0025
GO:0010941	regulation of cell death	873	16	2.2	0.00271
GO:0032990	cell part morphogenesis	284	8	3.3	0.00284
GO:0030029	actin filament-based process	286	8	3.3	0.00296
GO:0000904	cell morphogenesis involved in differentiation	290	8	3.3	0.00322
GO:0000165	MAPKKK cascade	294	8	3.2	0.0035

GO:0009968	negative regulation of signal transduction	120	5	4.9	0.00351
GO:0023057	negative regulation of signaling process	122	5	4.9	0.00377
GO:0031098	stress-activated protein kinase signaling cascade	122	5	4.9	0.00377
GO:0031175	neuron projection development	307	8	3.1	0.00454
GO:0021955	central nervous system neuron axonogenesis	13	2	18.2	0.00523
GO:0001933	negative regulation of protein amino acid phosphorylation	42	3	8.3	0.00535
GO:0051493	regulation of cytoskeleton organization	134	5	4.4	0.00561
GO:0008333	endosome to lysosome transport	14	2	16.7	0.00606
GO:0070303	negative regulation of stress-activated protein kinase signaling cascade	14	2	16.7	0.00606
GO:0010648	negative regulation of cell communication	324	8	2.9	0.00625
GO:0030326	embryonic limb morphogenesis	87	4	5.4	0.00634
GO:0035113	embryonic appendage morphogenesis	87	4	5.4	0.00634
GO:0035467	negative regulation of signaling pathway	259	7	3.2	0.00646
GO:0043254	regulation of protein complex assembly	88	4	5.3	0.0066
GO:0006917	induction of apoptosis	329	8	2.9	0.00683
GO:0012502	induction of programmed cell death	330	8	2.9	0.00695
GO:0070302	regulation of stress-activated protein kinase signaling cascade	90	4	5.3	0.00714
GO:0043506	regulation of JUN kinase activity	47	3	7.5	0.00733
GO:0051128	regulation of cellular component organization	634	12	2.2	0.00734
GO:0032535	regulation of cellular component size	334	8	2.8	0.00746

GO:0015782	CMP-sialic acid transport	1	1	100	0.00848
GO:0035408	histone H3-T6 phosphorylation	1	1	100	0.00848
GO:0035107	appendage morphogenesis	96	4	4.9	0.00893
GO:0035108	limb morphogenesis	96	4	4.9	0.00893
GO:0010639	negative regulation of organelle organization	97	4	4.9	0.00926
GO:0051258	protein polymerization	99	4	4.8	0.00993
